# Fetomaternal Outcomes of Severe Anemia in Pregnancy: A Prospective Observational Study

**DOI:** 10.7759/cureus.73834

**Published:** 2024-11-17

**Authors:** Chandrashekhar Shrivastava, Ruchi Bansal, Nitesh Goyal

**Affiliations:** 1 Obstetrics and Gynaecology, All India Institute of Medical Sciences, Raipur, Raipur, IND; 2 Pulmonary, Critical Care and Sleep Medicine, All India Institute of Medical Sciences, Raipur, Raipur, IND

**Keywords:** fetomaternal outcomes, maternal mortality, maternal & neonatal outcomes, neonatal mortality, severe anemia in pregnancy

## Abstract

Introduction

Anemia during pregnancy can lead to poor pregnancy outcomes, increasing maternal, fetal, and neonatal morbidity and mortality. Timely detection and management can lead to improved pregnancy outcomes.

Objective

To study various fetomaternal outcomes in severe anemia during pregnancy.

Methods

It is a prospective observational study conducted on 102 pregnant females, who presented with severe anemia (hemoglobin (Hb) < 7 gm/dL) between February 2020 and March 2021.

Results

In this study, 50% of patients presented before term (<37 weeks of gestation). Patients predominantly belonged to the rural population (n = 71, or 69.61%), the majority were multigravidas (81.37%), and 56.86% belonged to the upper-lower socioeconomic class. The mean age at presentation was 26.55 ± 4.99 years. Among maternal outcomes, abruption was seen in 7.84% of patients, post-partum hemorrhage (PPH) in 14.71%, sepsis in 3.92%, prolonged hospitalization in 48.04%, intensive care unit (ICU) admission in 4.90%, and maternal mortality in one patient. Additionally, 82.35% of patients went into spontaneous labor, and 76.47% of patients delivered vaginally. Among fetal and neonatal outcomes, 50% of neonates were premature, 62.75% were low birth weight (LBW), 42.16% were small for gestational age (SGA), and 22.55% of neonates were admitted to the neonatal ICU, of which two neonates expired. Stillbirth was noted in six (5.88%) babies. When the studied population was divided on the basis of the severity of anemia into two groups (Group A: very severe anemia with Hb < 4 gm/dL and Group B: severe anemia with Hb ≥ 4 gm/dL but <7 gm/dL), most of the outcomes were much worse for group A, with a statistically significant difference.

Conclusion

Not only can very severe anemia (Hb < 4 gm/dL), but also severe anemia (Hb ≥ 4 gm/dL but <7 gm/dL), adversely affect both maternal and fetal outcomes if not diagnosed and optimized on time.

## Introduction

Pregnancy is a physiological state characterized by enormous dynamic changes required for fetal growth and development. It is considered a state of stress, whereby these physiological changes unmask underlying medical disorders that were previously silent. Medical disorders interfere with the physiological adaptations of pregnancy and cause poor pregnancy outcomes [1].

Anemia during pregnancy is one of the major disorders associated with numerous maternal and fetal complications. It decreases the woman’s reserve to tolerate bleeding, either during or after childbirth, and makes her prone to infections. Anemia during pregnancy is found to be associated with an increased risk of intrauterine growth restriction, premature delivery, low birth weight (LBW), and maternal and neonatal mortality [2].

A meta-analysis of 52 studies showed that the prevalence of anemia among pregnant women is 37% globally [3]. The condition is so prominent in Southeast Asian countries that they account for half of all global maternal deaths due to anemia. India contributes to about 80% of maternal deaths due to anemia in South Asia. According to results from the National Family Health Survey (NFHS)-5, there is an increase in the prevalence of anemia among pregnant women in India by 1.4% when compared to NFHS-4 data [4]. Early detection and timely intervention with a multidisciplinary approach, including obstetrician, neonatologist, anesthetist, and skilled nursing care, is fundamental to the reduction of both maternal and neonatal mortality and morbidity.

This research is an attempt to assess the prevalence of anemia, along with its different types of presentations and complications, to help improve various maternal and neonatal outcomes. This is in alignment with effective planning and management of such pregnancies, so that better surveillance and obstetric intervention can be done at the right time, with better integration at each level of the health system, to improve the prognosis and outcomes of such pregnancies in Indian settings.

## Materials and methods

This is a prospective observational study conducted on 102 pregnant females who presented with severe anemia, between February 2020 and March 2021. This study was conducted in the Department of Obstetrics and Gynaecology of a tertiary care hospital, after approval from the Institutional Ethical Committee.

Sample size calculation

The sample size was calculated as per the following equation, where ‘N’ is the sample size of the single study group; ‘p’ is the prevalence of disease for the group; ‘z’ is the confidence level according to the standard normal distribution (for a level of confidence of 95%, z = 1.96; for a level of confidence of 99%, z = 2.575); and ‘d’ is the precision (tolerated margin of error).

\[ N = \frac{z^2 \cdot p(1 - p)}{d^2} \]

As in the majority of studies, p-values are considered significant below 0.05 (level of confidence of 95%); hence, 1.96 was used in the formula. According to the previous year's hospital records, it was found that the prevalence of severe anemia among pregnant patients was 7.1%. It was estimated that a sample size of 102 was needed to demonstrate a 7.1% prevalence of severe anemia among pregnant patients, with a 95% confidence interval and 5% precision.

Sampling

All consecutive pregnant women with hemoglobin (Hb) <7 gm/dL, admitted to the labor room or ward, were enrolled for the study after obtaining proper consent and explaining the study protocol. Pregnant females with severe anemia due to acute bleeding, or with preexisting medical comorbidities like hypertension, diabetes, hypothyroidism, and cardiac disease, were excluded from the study. A detailed history, examination, and necessary investigations were carried out on the selected subjects. Various maternal, fetal, and neonatal outcomes were noted.

Definitions

Severe anemia is defined as Hb concentration <7 gm/dL [5], while very severe anemia is defined as Hb concentration <4 gm/dL [5]. Prematurity is defined as gestational age <37 weeks [6], LBW as babies weighing <2.5 kg at birth [7], and small for gestational age (SGA) is defined as birth weight below the 10th percentile for the gestational age [8]. Prolonged hospitalization in our study was defined as a stay in the hospital for more than five days.

Statistical analysis

The data collected in this study were entered into a Microsoft Excel (Microsoft® Corp., Redmond, WA, USA) spreadsheet for initial organization. For preliminary descriptive analysis, frequencies and percentages were calculated to summarize the qualitative (categorical) variables. To further analyze the data, we used IBM SPSS Statistics for Windows, Version 21 (Released 2012; IBM Corp., Armonk, NY, USA), for statistical computations. Descriptive statistics were used to express the distribution of categorical variables as frequencies and percentages.

To assess associations between categorical variables, we employed two different tests: the Chi-square test and Fisher’s exact test. The Chi-square test was used when the expected count of each category was sufficiently large (typically, expected counts ≥5). In cases where the expected counts were <5, we applied Fisher’s exact test, which is more suitable for small sample sizes and ensures more accurate results when expected cell counts are low.

The level of statistical significance was determined by the p-value. A p-value of <0.05 was considered statistically significant, indicating a less than 5% likelihood that the observed association occurred by chance. Conversely, a p-value of >0.05 was considered non-significant, suggesting that the evidence was insufficient to reject the null hypothesis.

## Results

A total of 102 patients presented with severe anemia during the study period of 13 months. The sociodemographic details of the studied subjects are depicted in Table 1. Of these, 50% of patients presented to us before term (<37 weeks of gestation), and most of them did not have any complaints related to anemia but were found to be anemic incidentally. Patients with severe anemia predominantly belonged to the rural population (n = 71, or 69.61%). The mean age at presentation was 26.55 ± 4.99 years, and 77 patients (75.49%) with severe anemia were less than 30 years of age. We found that severe anemia was more prevalent in multigravida (n = 83, or 81.37%) as compared to primigravida (n = 19, or 18.63%). Most of the patients belonged to the upper-lower socioeconomic class according to the modified Kuppuswamy scale (n = 58, or 56.86%). Only 19 patients (18.63%) were symptomatic, and the rest were asymptomatic at the time of presentation.

**Table 1:** Sociodemographic characteristics of the study population (n = 102)

Variables	N (%)
Period of gestation at the time of presentation	
<20 weeks	1 (0.98%)
20-23.6 weeks	1 (0.98%)
24-27.6 weeks	1 (0.98%)
28-31.6 weeks	3 (2.94%)
32-36.6 weeks	45 (44.12%)
>37 weeks	51 (50%)
Residence	
Rural	71 (69.61%)
Urban	31 (30.39%)
Age	
<25 years	44 (43.14%)
25-30 years	33 (32.35%)
30-35 years	19 (18.63%)
≥35 years	6 (5.88%)
Gravida	
Primigravida	19 (18.62%)
Multigravida	83 (81.37%)
Socioeconomic status	
Upper	1 (0.98%)
Upper middle	4 (3.92%)
Lower middle	21 (20.59%)
Upper lower	58 (56.86%)
Lower	18 (17.65%)

Table 2 describes the various maternal, fetal, and neonatal outcomes in the studied population. Among maternal outcomes, abruption was seen in eight (7.84%) patients, post-partum hemorrhage (PPH) in 15 (14.71%), sepsis in four (3.92%), prolonged hospitalization in 49 (48.04%), intensive care unit (ICU) admission in five (4.90%), and maternal mortality in one patient. Most of the patients went into spontaneous labor (n = 84, or 82.35%). Of these, 78 (76.47%) patients delivered vaginally. Among fetal and neonatal outcomes, 51 (50%) neonates were premature, 64 (62.75%) were LBW, and 43 (42.16%) were SGA. Additionally, 23 (22.55%) neonates were admitted to the neonatal ICU (NICU), two of whom expired. Stillbirth was noted in six (5.88%) babies.

**Table 2:** Maternal, fetal, and neonatal outcomes among the study subjects (n = 102) PPH: Post-partum hemorrhage; ICU: Intensive care unit; SGA: Small for gestational age; NICU: Neonatal intensive care unit

Outcomes	N (%)
Maternal outcomes	
Abruption	8 (7.84%)
PPH	15 (14.71%)
Sepsis	4 (3.92%)
Prolonged hospitalization	49 (48.04%)
ICU admission	5 (4.90%)
Mortality	1 (0.98%)
Induced labor	18 (17.64%)
Spontaneous labor	84 (82.35%)
Vaginal delivery	78 (76.47%)
Cesarean delivery	24 (23.53%)
Fetal and neonatal outcomes	
Prematurity	51 (50%)
Low birth weight	64 (62.75%)
SGA	43 (42.16%)
NICU admission	23 (22.55%)
Mortality	2 (1.96%)
Stillbirth	6 (5.88%)

When the studied population was divided on the basis of the severity of anemia into two groups (Group A: very severe anemia with Hb < 4 gm/dL, and Group B: severe anemia with Hb ≥ 4 gm/dL but <7 gm/dL), most of the patients came under Group B (n = 76, or 74.5%). Patients with very severe anemia (Group A) did not give us sufficient time for Hb correction, as most of them went into spontaneous labor, compared to patients with severe anemia (Group B) (p-value = 0.032). More patients with severe anemia (Group B) underwent cesarean section (n = 23, or 30.36%) with a statistically significant difference (p-value = 0.006). Maternal sepsis was more common in Group A (n = 3, or 11.54%) compared to Group B (n = 1, or 1.32%) with a statistically significant difference (p-value = 0.05). No statistically significant difference could be found for the rest of the variables, including abruption, PPH, ICU admissions, maternal mortality, and prolonged hospitalization (Table 3).

**Table 3:** Comparison of maternal outcomes among two groups (n = 102) Hb: Hemoglobin; PPH: Post-partum hemorrhage; ICU: Intensive care unit

Criteria	Group A (N = 26), Hb < 4 gm/dL	Group B (N = 76), Hb ≥ 4 gm/dL	p-value
	N (%)	N (%)	
Spontaneous labor	25 (96.15)	59 (77.63)	0.032
Cesarean delivery	1 (3.85)	23 (30.26)	0.006
Abruption	0 (0)	8 (10.53)	0.11
PPH	2 (7.69)	13 (17.11)	0.343
ICU admission	2 (7.69)	3 (3.95)	0.56
Maternal sepsis	3 (11.54)	1 (1.32)	0.05
Maternal mortality	0 (0)	1 (1.32)	1
Prolonged hospitalization	15 (57.69)	39 (51.31)	0.574

Table 4 depicts that LBW and SGA were found to be more common in neonates born to mothers with very severe anemia (Group A) (84.62% and 65.38%, respectively) as compared to those born to mothers with severe anemia (Group B) (55.26% and 32.89%, respectively), with statistically significant differences (p-value = 0.009 and 0.004, respectively). Although prematurity (57.69%) and NICU admission (26.92%) were more prevalent in neonates born to mothers with very severe anemia (Group A), no statistically significant difference could be established.

**Table 4:** Comparison of fetal and neonatal outcomes among the two groups (n = 102) Hb: Hemoglobin; SGA: Small for gestational age; NICU: Neonatal intensive care unit

Criteria	Group A (N = 26), Hb < 4 gm/dL	Group B (N = 76), Hb > 4 gm/dL	p-value
	N (%)	N (%)	
Prematurity	15 (57.69)	36 (47.37)	0.363
Low birth weight	22 (84.62)	42 (55.26)	0.009
SGA	17 (65.38)	25 (32.89)	0.004
Stillbirth	1 (3.85)	5 (6.58)	1
NICU admission	7 (26.92)	16 (21.05)	0.536
Mortality	0 (0)	2 (2.63)	0.403

## Discussion

Anemia during pregnancy is one of the important factors associated with a number of maternal and fetal complications, including sepsis, maternal mortality, neonatal prematurity, and LBW [2,9]. This generates much of our interest in its prevalence and its effect on fetomaternal outcomes, so as to plan effective intervention strategies at the right time, making the journey of pregnancy safer and a more cherished experience.

Most of the anemic patients present to the health care facility when they go into labor, and anemia among them gets detected incidentally [10]. Similar findings were observed in our study as well, since only 18.63% of patients who presented to us were symptomatic for anemia. A significant number of cases presented between 32 and 37 weeks of gestation (n = 45, or 44.12%). Out of all patients who were having symptoms related to anemia, 58.82% were between 32 and 37 weeks of gestation. This can be explained by the fact that 32 to 34 weeks of gestation is a high-risk period for pregnant patients to develop decompensation and present to the hospital [11].

It was also observed in our study that patients with severe anemia predominantly belonged to the rural population (n = 71, or 69.61%). This considerable difference is attributed to multiple factors, such as lack of awareness, poor nutrition, lower socio-economic status, multiparity, and poor accessibility to healthcare facilities in rural populations [12]. In a study conducted by Devi et al., they also observed that the majority of patients belonged to rural areas (84%), and very few belonged to urban areas [13].

Severe anemia was most prevalent in patients less than 30 years of age, with 75.49% of patients in our study. The mean age of presentation was 26.55 ± 4.99 years. This can be explained by the early age of marriage and childbearing in the study population. Similar observations were also made by Shi et al., as the majority of pregnant females were less than 30 years old, and the mean age was 29.42 ± 4.87 years in their study [14]. Similarly, Suryanarayana et al. also reported that 66.1% of patients in their study were between 21 and 30 years of age [9]. It was also observed in our study that severe anemia was more prevalent in multigravida (n = 83, or 81.37%) than in primigravida. Similar results were also stated by Devi et al., as they observed in their study that the majority of the anemic pregnant women were multigravida (53.5%) [13]. In our study, it was found that most of the patients with severe anemia belonged to the upper-lower socioeconomic class according to the modified Kuppuswamy scale (n = 58, or 56.86%). Patients in the upper-middle and upper classes were minimal, combining to 4.9%. According to Devi et al., the majority of the patients (88.82%) belonged to lower socioeconomic status [13].

We found that, among maternal outcomes, abruption was seen in eight (7.84%) patients, PPH in 15 (14.71%), sepsis in four (3.92%), prolonged hospitalization in 49 (48.04%), ICU admission in five (4.90%), and maternal mortality in one patient. Most of the patients went into spontaneous labor, i.e., 84 (82.35%), while labor was induced in the rest (17.64%). Of the total, 76.47% of patients delivered vaginally, and the remaining 23.53% had cesarean delivery. Among fetal outcomes in our study, 51 (50%) neonates born to anemic patients were premature, 64 (62.75%) were LBW, and 43 (42.16%) were SGA. Additionally, 23 (22.55%) neonates were admitted to the NICU, of which two (1.96%) expired. Stillbirth was noted in six (5.88%) babies.

Similar findings were observed in a study conducted by Smith et al., where they found that anemic women had longer hospitalization durations and more antenatal admissions. They also observed that anemia was associated with neonatal prematurity, SGA, low five-minute APGAR score, neonatal death, and perinatal death [15]. Anadkat and Goswami stated in their study that the most common complications associated with anemia in pregnancy were PPH, pulmonary edema, and surgical site infections. The majority of patients delivered vaginally. LBW was reported in 67.46% of newborns in their study, and 39.75% of newborns had moderately abnormal APGAR scores, leading to increased NICU admissions [16]. Bansal and Singhal also found that there was an increased incidence of preterm delivery, PPH, and mortality in anemic pregnant patients. Among the adverse fetal outcomes, they reported an increased incidence of intrauterine deaths, intrauterine growth restriction, NICU admission, and LBW among the newborns of anemic patients [17]. Hansda et al. reported that preterm labor (30%), PPH (9.6%), abruption (7%), sepsis (9.8%), and maternal mortality (1.27%) were more common in pregnant patients with anemia [18]. Also, newborns of such patients were found to have increased complications like prematurity (11.3%) and intrauterine fetal death (2.7%).

In our study, we found that pregnant patients with very severe anemia (Hb < 4 gm/dL) went into spontaneous labor, as compared to patients with severe anemia (Hb ≥ 4 gm/dL but <7 gm/dL), with a statistically significant difference (p-value = 0.032). More patients with Hb ≥ 4 gm/dL underwent cesarean section (n = 23, or 30.36%), as compared to the other group, with a statistically significant difference (p-value = 0.006). From our study, it can be interpreted that patients who are more severely anemic tend to go into spontaneous labor and end up delivering vaginally, without giving us sufficient time to achieve the target Hb. Similar results were published by Klebanoff et al., who stated that the presence of anemia in pregnant females leads to spontaneous preterm deliveries [17,19]. Patients with severe anemia (Hb ≥ 4 gm/dL but <7 gm/dL) required induction of labor after correction of their anemia and ended up having a cesarean section, attributed to the risks created by the inducing agents [20]. We also found that very severely anemic patients (Hb < 4 gm/dL) had a statistically more significant incidence of maternal sepsis when compared to severely anemic patients with Hb ≥ 4 gm/dL (p-value = 0.05). Although ICU admissions and prolonged hospitalization were more common in patients with very severe anemia (Hb < 4 gm/dL) as compared to the other group, there was no statistically significant difference.

In our study, we observed that LBW (84.62% vs. 55.26%) and SGA (65.38% vs. 32.89%) were statistically more common in newborns of very severely anemic patients (Hb < 4 gm/dL) when compared to patients with severe anemia (Hb ≥ 4 gm/dL but <7 gm/dL). Although prematurity (57.69% vs. 47.37%) and NICU admission (26.92% vs. 21.05%) were more prevalent in anemic patients with very severe anemia (Hb < 4 gm/dL), as compared to the other group, the difference between them was not statistically significant. Kumari et al. observed in their study that anemia in pregnancy was strongly associated with preterm birth (p-value ≤ 0.0001) and LBW (p-value = 0.0003). They also observed that preterm birth and LBW were dependent on the stratification of anemia, with the strongest association found between severe anemia and these parameters [21]. Similar results were reported in a study conducted by Lone et al., where they studied the effects of anemia on pregnancy outcomes. They observed that the risk of preterm delivery and LBW among anemic pregnant patients was 4 and 1.9 times higher, respectively, when compared to nonanemic pregnant patients [22].

Geelhoed et al. also compared pregnant women with severe anemia and non-anemic pregnant women. They found that anemic pregnant women had an increased risk of maternal death (3.18% vs. 0%). Regarding fetal outcomes, they concluded that perinatal mortality and LBW were more common in newborns of severely anemic pregnant patients [23]. Shi et al., in their study, stratified anemia into mild, moderate, and severe, and compared maternal and fetal outcomes among these groups. They found that moderate and severe anemia was associated with statistically significant increased risks of maternal shock, ICU admission, maternal death, fetal growth restriction, and stillbirth when compared with mild anemia [14]. Smith et al. also observed that pregnant women with severe anemia had significantly higher odds of prolonged hospital stay (less than seven days), PPH, preterm birth, SGA birth, stillbirth, low five-minute APGAR score, and perinatal death compared to women with mild and moderate anemia [15].

In the literature reviewed, only a limited number of studies exist comparing maternal and fetal outcomes among pregnant patients with mild, moderate, and severe anemia. On the other hand, no studies exist comparing these outcomes between severely anemic and very severely anemic pregnant patients.

A key limitation of the study is the limited number of cases included. Additionally, several factors - such as the etiology of anemia, dietary habits, previous treatment, and use of prenatal iron - were not systematically recorded, as they were not part of the original study protocol. Assessing the etiology of anemia could have provided valuable insights and potentially enhanced the interpretation of outcomes.

We would like to highlight a significant strength of this study: early detection and management of anemia. This center has established protocols for the timely identification and correction of anemia, which have likely contributed to improved outcomes for the patients involved. The proactive management of anemia in this setting is a notable strength and may have helped mitigate the severity of anemia and its associated complications.

## Conclusions

Our study throws considerable light on the devastating effects of extreme anemia (Hb < 7 gm/dL) on maternal and neonatal outcomes. Women with severe anemia had more complications, including PPH, abruption, sepsis, and longer hospital stays. Additionally, very severe anemia (Hb < 4 gm/dL) had even more adverse effects, with higher incidences of spontaneous labor, maternal sepsis, and ICU admissions. These findings advocate the need to recognize anemia as a serious risk during pregnancy, especially in populations with limited access to health services. Early detection and management are crucial strategies to prevent the worsening of maternal conditions and to reduce pregnancy-related morbidity and mortality.

As for neonatal outcomes, the current study demonstrates that prematurity, LBW, SGA, and NICU admissions are significantly higher in infants born to mothers with severe anemia. Such disorders have long-term implications for neonatal survival and development. Given that a significant proportion of patients with anemia came from rural and lower socioeconomic backgrounds, improving access to prenatal care and nutritional support in these populations is essential. A combined approach of obstetricians, neonatologists, and public health experts is crucial to curbing the effects of severe anemia in pregnancy. It is also possible to improve outcomes for both the mother and fetus through timely interventions, such as the administration of iron or blood when needed, making pregnancy less risky for at-risk populations.
